# Brazilian National School Feeding Program: A Review with Content Analysis of Social Documents Using MaxQda^®^ Software

**DOI:** 10.3390/nu17213436

**Published:** 2025-10-31

**Authors:** Ygraine Hartmann, Denise Bomtempo Birche de Carvalho, Renata Puppin Zandonadi, Raquel B. A. Botelho, Rita de Cassia Coelho de Almeida Akutsu

**Affiliations:** 1 Diretoria de Acessibilidade do Decanato de Assuntos Comunitários, University of Brasília, Campus Universitario Darcy Ribeiro, Brasilia 70910-900, Brazil; 2 Department of Social Work, Institute of Human Sciences, University of Brasília, Campus Universitario Darcy Ribeiro, Brasilia 70910-900, Brazil; denisebbcarvalho@gmail.com; 3 Department of Nutrition, Faculty of Health Sciences, University of Brasília, Campus Universitario Darcy Ribeiro, Brasilia 70910-900, Brazil; renatapz@unb.br (R.P.Z.); raquelbotelho@unb.br (R.B.A.B.); ritaakutsu@unb.br (R.d.C.C.d.A.A.)

**Keywords:** human right to adequate food (HRAF), food and nutrition security, national school feeding program, public health, nutrition policy, Brazil

## Abstract

**Background**: Food and Nutrition Security (FNS) policies in Brazil aim to ensure a broad spectrum of rights and were formulated based on the complex relationship among the State, Society, and Market in the capitalist order. The human right to adequate food (HRAF) is reflected in the guidelines of the National School Feeding Program (Programa Nacional de Alimentação Escolar—PNAE), which is implemented and monitored by the National Education Development Fund (*Fundo Nacional de Desenvolvimento da Educação—FNDE*). The PNAE consolidates part of the strategies adopted by the Brazilian State to combat hunger among children and adolescents and promote healthy habits by offering food in schools. However, there is no recent evaluation on the aspects of management, financing, and monitoring of the PNAE in the Brazilian Federal District. **Objective**: This study aims to contribute to the debate on health promotion and the right to adequate food by analyzing documents and legislation in force until 2024 related to the PNAE, as well as those that maintain a correlation with food and nutritional security policies in Brazil, verifying the scope and guarantee of rights and their applicability based on a critical analysis of the selected database. **Methods**: Brazilian official documents and legislation related to FNS and school meals were reviewed for inclusion in the database. The historical-dialectical materialist method was employed for content analysis, and the analyzed documents underwent a critical reading and the coding process, grouping common themes, utilizing MaxQda^®^ software for support. **Results**: The word cloud (20 words) shows that the document groups emphasize food as a right, with 6038 occurrences found and relating to the “rights” approach with 2365 occurrences found, highlighting words related to FNS policies. Public actions to achieve health and food supply were expressed through the words “public” (381 occurrences) and “DHAA” (510 occurrences). The code cloud highlights social control as the most frequently attributed code in the set of documents, with 105 codified segments, indicating that democratic control and societal participation are crucial for achieving the PNAE’s objectives. **Conclusions**: The analyses underscored the importance of social control, as evidenced by the exploration of the research corpus and demonstrated in the code cloud. The role of monitoring and social control falls to the School Feeding Council (CAE), being the body responsible for verifying compliance with the Program’s objectives, enabling the adoption of timely measures to correct the PNAE’s course and contribute to achieving its objectives.

## 1. Introduction

Considered the oldest social policy in the field of Food and Nutrition Security in Brazil, the National School Feeding Program (*Programa Nacional de Alimentação Escolar*—PNAE), created in the 1950s, arose from the need to ensure food for students throughout basic education in public schools [1].

Nowadays, PNAE’s objective is to meet the nutritional needs of students during their time in the classroom, thereby contributing to their growth, development, learning, and academic performance, and promoting healthy eating habits [2], and it contributes significantly to the fight against hunger and malnutrition, positioning itself as one of the Food and Nutrition Security (FNS) policies. Understanding the PNAE in the Brazilian context, placing it in the historical context of its trajectory and its entirety, is fundamental to understanding the current structure of the program and its future challenges. The PNAE is a wide-ranging program, and according to the National Fund for the Development of Education, it has a budget of R$5,461,907,292.00 to serve approximately 38,531,387 students enrolled in basic education in the Federal, State, District, and Municipal networks of Brazil [3].

In a country like Brazil, founded on colonial exploitation, understanding the roots that sustain the Brazilian economic system and the consequences of social inequalities is essential to discuss the importance of social programs linked to Food and Nutrition Security (FNS). Additionally, it is crucial to assess the necessary steps to address such inequalities. In addition, it is necessary to determine the format of Brazilian agricultural production, its objectives, insertion, and the construction of feeding and social behavior.

Governments, organizations, and scientists have recently discussed hunger and its consequences. In this sense, addressing it by discussing its contradictions within capitalist society and advances in FNS at national and international levels is urgent to overcome social inequalities and guarantee the Human Right to Adequate Food (HRAF).

Thus, this research is based on two basic assumptions: (1) the social and feeding issues are inseparable and are directly intertwined with the socio-historical and socioeconomic roots of Brazilian society; (2) food at school plays a crucial role in ensuring the FNS of Brazilian children and the formation of healthy eating habits.

These analyses must consider the relationships between the State, the Market, and Society, considering that each exerts force to meet its demands. According to Pereira [4], over the last three decades, the global scenario has been shaped by two trends of transformation: the consolidation of market mechanisms and democracy, which are considered the basis for structuring societies. To this end, it is necessary to observe the entirety of the issue in the capitalist order. The incessant pursuit of accumulation, profit generation, and private property has a direct impact on the most disadvantaged layers of society, regardless of the consequences it has on the population’s health and the formation of dietary patterns.

For a long time, poverty was mistakenly treated as a natural condition of people who, in special situations, were left helpless and deserving of state support. In this sense, poverty was viewed as an imbalance in economic relations, or even, in the Malthusian conception, the capacity to produce food would not be sufficient to meet the needs of the entire population [5]. In this sense, social policies would not be able to overcome the inequalities between classes; however, they have always been an important field of dispute for guaranteeing subsidies that alleviate existing disparities. Without intending to hierarchize social problems, hunger is one of the most serious consequences, an expression of current social inequality, characterized by discontinuous and fragmented social food and nutrition policies [6,7].

Regarding school meals, which in Brazil corresponds to the provision of a meal that meets, at least, 10% of the nutritional needs of a student for 24 h, when it was implemented, its objective was to provide food to elementary school students, which corresponded to the first four years of formal education in Brazil. At that time, the school meals also aimed to alleviate conflicts with the working class, whose children were in the educational system. In addition, in Brazil’s late industrialization, a minimum wage was established, balanced meals were offered, and food supplies were guaranteed to formal workers in urban-industrial centers. These actions were intended to alleviate tensions and incorporate the values and ideology that characterize industrial capitalist labor relations and processes, regardless of the consequences of such actions [8].

Thus, throughout the construction of the Brazilian economic model, school meals were generally standardized in terms of supply, with centralized acquisition and incentives for the consumption of industrialized products. These characteristics were consolidated to such an extent that even today, we find remnants of this acquisition format and, consequently, its influence in school meals [9].

Among the main changes in schoolchildren’s eating patterns is the replacement of *in natura* or minimally processed plant-based foods (e.g., rice, beans, cassava, potatoes, and other vegetables) with industrialized preparations, including canned beans. These changes in food consumption patterns were, and still are, intense in Brazil, with consequences including nutrient imbalances and excessive calorie intake [10].

The importance of discussing this topic lies in the dispute between the strong economic interests that permeate the production and marketing of food versus the responsibility of providing natural, nutritious, and healthy food. Strengthening the areas of study in the field of nutrition can directly contribute to the formulation and consolidation of FNS policies. Thus, not only the quantity, but also the quality of the food made available within the scope of policies that involve the food supply should be analyzed based on the understanding that the food offered can be a factor in promoting health or disease, with publications featuring the most diverse analyses.

Given this scenario and the inexistence of a recent evaluation that considered the aspects of management, financing and monitoring of the PNAE in the Brazilian Federal District, investigating FNS strategies within the scope of public food and nutrition policies becomes essential to successfully promote their objectives, as they should contribute to alleviating the problems related to the nutritional status of target populations. In this context, this study aims to contribute to the debate on health promotion and the right to adequate food by analyzing documents and legislation in force until 2024 related to the PNAE, as well as those that maintain a correlation with food and nutritional security policies in Brazil, verifying the scope and guarantee of rights and their applicability based on a critical analysis of the selected database. The results were generated through the analysis of word clouds and code clouds, created with the MaxQda^®^ 2020 software.

## 2. Materials and Methods

This qualitative study utilized secondary data collection from all official Brazilian government documents, including laws, Resolutions, and booklets from institutional bodies implementing food and nutritional security policies, as well as drew on the Council of Nutritionists, as described in Table 1, which remains in force until 2024.

Qualitative research in the social sciences is concerned with the universe of meanings, motives, aspirations, beliefs, values, and attitudes, which cannot be reduced to the operationalization of variables, corresponding to a deeper field of relationships [10] (p. 21).

The Content Analysis technique was employed in its heuristic function in the thematic modality, without presenting provisional hypotheses, to develop and enrich the analyses related to the objectives outlined in this research. This technique provides the researcher with a creative process for interpreting secondary data, in light of theory, practice, and manifest content [10]. Content analysis enriches exploratory efforts and increases the propensity for discovery [11].

Following the methodology proposed by Bardin [12], the pre-analysis of the selected documents was conducted to establish the research corpus. The corpus is considered the set of documents that are submitted to the analytical procedures. Its constitution must respect the rules of exhaustiveness, representativeness, homogeneity and relevance [12].

To carry out the content analysis, the data collected for this study were subjected to qualitative analysis using MaxQda^®^ 2020 software, an academic software for analyzing qualitative data and mixed research methods. MaxQda^®^ is a tool for researchers working with qualitative data, offering several features that facilitate the organization, analysis, and interpretation of data, contributing to more efficient research and more significant results.

### 2.1. Search and Selection of Secondary Data

The secondary data used in this research are part of a range of Public Policies, Legislation, standards, and regulations that deal with the design, management, financing, and monitoring of the PNAE, as well as documents from institutions involved in its execution, monitoring, and democratic control, as well as data from the public transparency portal.

To develop the theoretical bases and discussions about the PNAE, free access databases (Scielo, LILACS, BVS) were used to search for articles, dissertations or theses, using the descriptors: “Human Right to Adequate Food”, “school feeding”, “Food and Nutrition Security”, “National School Feeding Program”, considering the period from 2006 to 2024. This period is justified due to the implementation of the Organic Law on Food and Nutrition Security-LOSAN in 2006, in Brazil, a year in which a profound effort could be detected both by the State and by social actors to consolidate the Right to Food and FNS, with legal frameworks that represented significant advances in this sense in Brazil [13].

### 2.2. Data Organization

The MaxQda^®^ software was used to organize the selected documents. Its objective is organization, although it is composed of unstructured, “open” activities, as opposed to the systematic exploration of documents [11] (p. 96). Data organization was based on the content analysis technique, which began with the import of textual documents that comprised the research corpus after the pre-analysis of the documents was completed. MaxQda is a software application for analyzing qualitative data and falls within the category of Computer-Assisted Qualitative Data Analysis (CAQDAS). Qualitative Data Analysis Software (QDAS) was employed in this study to support the analysis of a large volume of documents [14].

To compile the database, documents related to the research objectives were included and grouped according to their theme and relevance to the objectives. Table 1 presents the set of documents inserted into the database.

**Table 1:** Secondary sources for database composition in MaxQda^®^ 2020 software.

Objective	Analysis/Publication Content	Brief Description of the Publication/Document
Identify the concepts that anchor the bases for guaranteeing FNS in Brazil, correlating them with the guidelines and actions within the scope of the PNAE *.	The Human Right to Adequate Food and SISAN ** [15].	Edition of the course on the Human Right to Adequate Food, carried out by the Ministry of Social Development and Fight against Hunger—MDS in partnership with the Brazilian Action for Nutrition and Human Rights—ABRANDH.
	Resolution No. 06, of 8 May 2020 [16].	Provides school meals to basic education students within the scope of the National School Feeding Program—PNAE (repeals Resolution No. 26 of 2013). Establishes criteria for acquiring food with federal resources transferred by the FNDE and minimum nutritional parameters for providing food to students in the public education system. It also deals with the role of nutritionists within the scope of the PNAE.
	Law No. 11,346, of 15 September 2006 [17].	Creates the National Food and Nutrition Security System—SISAN ** to ensure the human right to adequate food and provides other measures.
	Law 11,947, of 16 June 2009 [18]	Provides for the provision of school meals and the Direct Cash to School Program to basic education students; amends Laws Nº 10,880 of 9 June 2004, 11,273 of 6 February 2006, 11,507 of 20 July 2007; repeals provisions of Provisional Measure Nº. 22178-36 of 24 August 2001, and Law Nº. 8.913, of 12 July 1994; and provides other measures.
	MDS Healthy Eating Manual [19].	This manual aims to provide adequate and healthy food to people participating in the PAA by qualifying the entities’ demand for food, aiming to guarantee the Human Right to Adequate Food (HRAF).
	National Food and Nutrition Policy, Ministry of Health [20].	The National Food and Nutrition Policy (PNAN) aims to improve food, nutrition, and health conditions, seeking to guarantee Food and Nutrition Security for the Brazilian population. Ordinance No. 2715, of 17 November 2011. Updates the National Food and Nutrition Policy.
	Decree 7272, of 25 August 2010 [21].	Regulates Law No. 11,346 of 15 September 2006, which creates the National Food and Nutrition Security System—SISAN to ensure the human right to adequate food, institutes the National Food and Nutrition Security Policy—PNSAN, establishes the parameters for the preparation of the National Food and Nutrition Security Plan, and provides other measures.
	CFN *** Resolution 465, of 23 August 2010 [22].	Provides for the duties of the Nutritionist, establishes minimum numerical reference parameters within the scope of the PNAE and provides other measures. Establishes the mandatory technical activities within the scope of the PNAE.
	Handbook for PNAE Counselors, 2017 [23].	Guidance material for developing social control activities within the scope of the PNAE. To encourage and improve the performance of the School Feeding Councils (CAE’s) in monitoring the School Feeding Program that extends throughout the national territory.
	National School Feeding Handbook, 2015 [24].	Guidance material for developing social control activities within the scope of the PNAE.

Source: Prepared by the authors based on the indicated references. * PNAE: National School Feeding Program, ** SISAN: National Food and Nutrition Security System, *** CFN: Federal Council of Nutritionists and Dietitians, MaxQda^®^ software-supported encoding.

After defining the research corpus presented in Table 1, the material exploration phase began, which is the longest and consists essentially of coding operations. MaxQda^®^ software provides a tool that allows you to search for keywords or concepts relevant to your research, optimizing the coding process.

According to Mellado et al. [25], the use of CAQDAS has optimized and facilitated the work of qualitative researchers, thanks to the development of software for qualitative analysis within the software support.

As a result, the analytical process has become more structured and reliable, enabling researchers to gain greater credibility in their work. Even so, using CAQDAS does not eliminate the need for the researcher to engage more closely with their object. In the material exploration phase, “the established corpus must be studied in depth to establish the Registration Units and Context Units. The context units are the ‘backdrop’ that gives meaning to the Analysis Units and serves as a unit of understanding to encode the Registration Unit” [26] (p. 43). Following this methodology, we sought to understand in which textual contexts (fragments) the registration units were inserted, to reveal possible approaches and focus in the analyzed documents.

Codes are the smallest units of analysis that express important characteristics of documents (potentially) relevant to the research question. The software allows the marking of coded segments, assigning distinct labels or colors, to facilitate data exploration. “After coding and reviews to align the information, interpretation and analysis are carried out” [27]. According to Bardin [12], “coding is the process by which raw data are systematically transformed and aggregated into units, which allow an accurate description of the pertinent characteristics of the content”. Coding presupposes the choice of units (cutting); enumeration (choosing counting rules); and classification and aggregation (choosing categories). The choice of codes must respond pertinently to the objectives of the analysis, and for this reason, a thematic or semantic cut was selected that characterizes each code.

The codes emerged from reading the material inserted in the database under analysis and express relevance insofar as they relate directly to the research objective. To begin coding the textual fragments of the documents that comprise the analysis corpus, codes were established a priori, defined primarily based on the research group’s experience and knowledge, and are presented in Table 2.

MaxQda^®^ software has coding functionality that allows the researcher to organize coded textual elements with themes and colors, facilitating the visualization and search for thoughts and theories linked to the project.

The definitions adopted guided the research to conceptually demarcate the aspects under analysis. During the process of reading and exploring the material, demarcations (cuts) were made with the Codes shown in Table 2.

Throughout the process of reading the explored materials, some registration units emerged, while others, defined a priori, were excluded because no occurrences were found in the analyses that related to the defined codes, or because they were classified under another coding deemed more appropriate.

The creation of initial codes occurred in an intuitive and exploratory manner. Intuitive (floating), as it considered the research group’s knowledge and experience in managing and operationalizing the PNAE, and exploratory, as the material’s progression necessitated the creation of new codes. The excluded codes were “Food Control and Regulation” and “Representation” and those included are presented in Table 3.

In this software (MaxQda), the coding process is performed by the user, and the tool assists in searching for sections of interest, as well as in separating and grouping them according to the criteria established by the researcher [27].

The next step was to identify themes. “Codes are building blocks of themes, larger patterns of meaning, supported by a central organizing concept—a shared central idea.” Codes were grouped in a way that maintained a meaningful relationship among them. “Themes provide a framework for organizing and reporting the researcher’s analytical observations, and reflect the researcher’s interpretive choices” [27].

After completing the thematic division phase, all documents were reanalyzed to verify the adequacy of code allocation. Through this continuous rereading process, it was possible to identify new textual fragments that could be incorporated into the sets of codes with each subsequent reading.

Categorization provides, through condensation, a simplified representation of the raw data [27]. In the specific case of the research in question, the categorization system was assigned by grouping the codes that fit into the themes explored, according to each previously defined objective. In this process, the conceptual title of each category was only determined at the end of the data analysis operation.

## 3. Results

The analysis of the research body was completed, yielding a total of 44 codes, grouped into two categories: Financing and Management and Supervision. During the analysis procedures, codes with similar meanings were grouped to form themes and subthemes, thereby organizing the code matrix.

**Figure 1:**
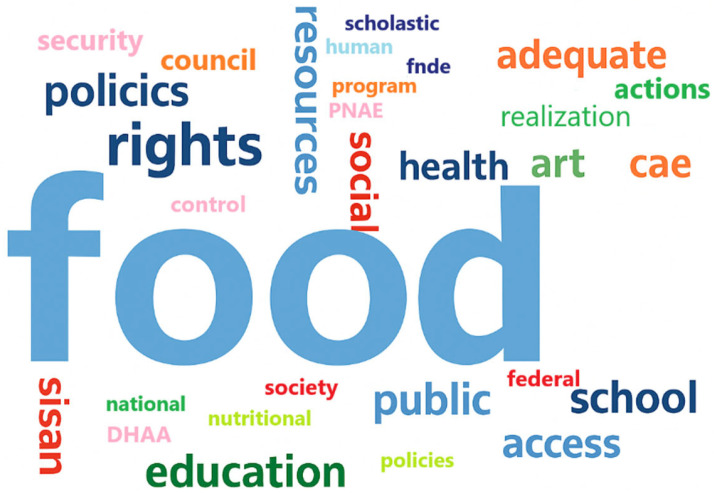
Word cloud—design of FNS and PNAE policies. Source: MaxQda^®^ Software.

In the thematic modality, this research employed content analysis, utilizing the techniques of “Word Cloud” and “Code Cloud”. The “Compare cases and groups” tool was used to access the coded segments for dynamic reading of the material and extraction of textual fragments of interest. The results obtained from the documents selected for the research corpus are presented in Figure 1. The research team used the software’s word cloud creation tool to provide a panoramic view of the keywords in the database, revealing the most frequently occurring terms. From this result, it was possible to determine what is most relevant within the context defined in the research corpus and the concepts described in Table 1. This figure utilizes a Word Cloud, considering a minimum frequency of 20 occurrences, without sensitivity to upper or lower case, and ignoring numbers.

The results relate to the design of the PNAE and other regulations associated with FNS, based on the documents selected for the research corpus, which aims to identify the concepts that anchor the basis for guaranteeing FNS in Brazil. These concepts are then correlated with the guidelines and actions within the scope of the PNAE in the Federal District, visually demonstrating that the defined research corpus reflects the purpose to be achieved, which is to guarantee the right to food, bringing many references to food, health, and rights within the scope of school meals.

Figure 1 a cloud of 30 words, highlights the words that received the greatest emphasis in the research corpus documents. Notably, the word “food” appears 6038 times, and the “rights” approach is represented 2365 times. Public actions to achieve health and food supply were expressed through the words “public” (381 occurrences) and “DHAA” (510 occurrences).

**Figure 2:**
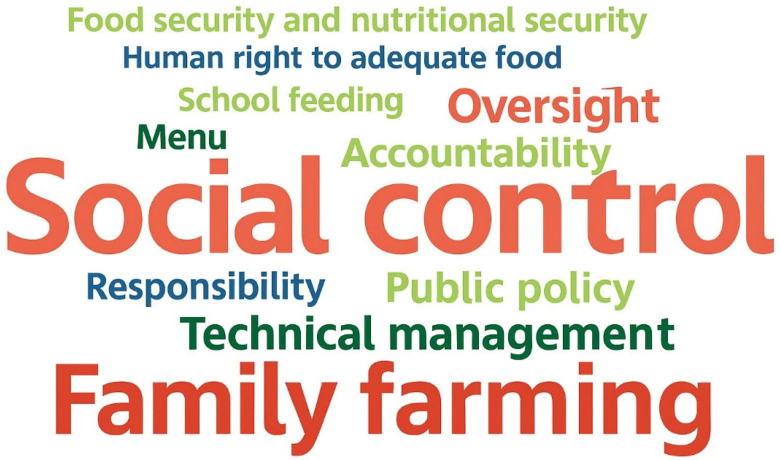
Code cloud—design of FNS and PNAE policies. Source: MaxQda^®^ software.

The documents on FNS policies that comprise the analysis corpus demonstrate technical and theoretical consistency, representing the search for FNS guarantees for school-age children served by the PNAE, to the extent that the terms identified as most frequent are directly related to the scope of rights associated with FNS in Brazil and the PNAE. It is also possible to note the absence in the cloud of words that relate to the monitoring or inspection of policies, that is, in this group of documents, there was no emphasis on the forms or tools for achieving the rights guaranteed, with only the word “control” standing out, with 327 occurrences. This result highlights the low frequency of terms that correlate with the monitoring and inspection of expected guarantees. From the same research corpus, a “Code Cloud” was also generated to verify the predominance of codes present in the same set of documents, as shown in Figure 2. The coding performed a priori (Table 2) adopted definitions to identify textual fragments that fit previously defined concepts, allowing the research team to interpret and assign such codes during the exploration of the material. For better visualization of the codes assigned during the exploration of the material, a Code Cloud is used, considering a minimum frequency of 20 occurrences of the code.

Although Social Control is not present in the Word Cloud in Figure 1, the Code Cloud presented in Figure 2, generated from the same documents, highlights this dimension of Public Policies, standing out with 105 coded segments. This means that although social control is not explicitly stated in the Word Cloud presented in Figure 1, this characteristic emerged from the coding process.

The coding process of the textual fragments from the set of analyzed legal documents identified fragments that, in some way, contextualized or represented social control actions. Thus, we can state that to ensure the right to food provided for in the consulted legislation, it is essential to have social control mechanisms in place.

Based on Figure 2, emphasis is placed on Family Farming (79 coded segments) to encourage healthy eating habits and the consumption of local and regional products. This incentive is an integral part of the guidelines of the SAN Policies, especially the PNAE, which has in its legal guidelines the obligation for public entities to acquire minimum percentages of products from family farming such as fruits, and vegetables, concerning the regional culture, also being a way of encouraging sustainability and the local economy.

Figure 2 illustrates a clear incentive to consume products from Family Farming, promoting healthy eating habits. This incentive is an integral part of the guidelines of the FNS Policies, especially the PNAE, which provides for the acquisition of fruits, vegetables, and pulses, as well as respect for regional culture, to encourage sustainability and the local economy.

Another aspect to be analyzed is the presence of actions related to the technical management of these Policies. From Figure 2, it can be inferred that there is a significant presence of the code “Technical Management” (61 coded segments), which represents the set of attributions related to the execution of policies, highlighting the importance of the institutional actors responsible for their execution in achieving their objectives.

Therefore, based on the analyses presented, it is concluded that social control is necessary to ensure the efficient technical management of Food and Nutrition Security programs, particularly the PNAE, to achieve the proposed objectives in the regulatory documents explored in this research.

## 4. Discussion

The results obtained achieved the objective of this research insofar as it was possible to identify that the main terms related to FSN stand out in the research corpus and from the exploratory and critical reading and the coding process it was possible to identify mechanisms of execution and search for quality in food, giving emphasis to the participation of society exercising social control.

The Brazilian State has a very voluminous and detailed set of laws regarding FNS policies. These documents reflect the establishment of fundamental rights related to the topic of food, and the PNAE is also included in this set of laws to ensure compliance with the program’s objectives.

The analysis verified the set of documents and laws that cover the topic of FNS in Brazil, also correlating it with the National School Feeding Program and its operationalization, which involves planning, execution, and monitoring stages.

The results obtained based on the exploration of Brazilian documents and legislation demonstrate a well-formulated legal framework regarding FNS and DHAA guarantees, which are directly linked to the execution of the PNAE policy. In this regard, PNAE managers must include in their purchases products that provide real food and reduce or eliminate industrialized products, valuing family farming and food and nutrition education, as shown in the code cloud (Figure 2).

Throughout the analyses conducted in Figure 1, it became clear that the documents analyzed did not bring emphasis on the PNAE’s qualitative control mechanisms, to assess the quality and quantity of products acquired by public entities, which may vary from region to region depending on the volume of resources provided locally, disregarding aspects of potential worsening of FNS conditions due to social inequalities in the different states of the Federation, or even between different administrative regions.

The actions of the School Feeding Council (CAE), when they have an adequate structure and representation, can be the most efficient way to adjust deviations in the conduct of the PNAE, since they can, through their monitoring and advisory role, indicate adjustments simultaneously with the execution of the Program, as demonstrated in the code cloud, the emphasis on social control. According to Silva and Muniz [37], in a study carried out with 146 Administrative Units (State and municipal), composed of 400 CAE in the state of Paraná, the greater the expertise of the CAE members, the greater the potential to execute routines that contribute to improving the conditions for offering healthy food, aligned with the principles of the PNAE [37]. The study carried out in Paraná by Silva and Muniz applies, in principle, to all Councils in all areas because knowledge or expertise has been indicated as a proxy indicator in several studies in Brazil and in other countries [37,38].

The actions of the CAE, when they have an adequate structure and representation, appear to be the most efficient way of adjusting deviations in the conduct of the PNAE, as they are able, through their monitoring and advisory role, to indicate adjustments during the execution of the Program.

According to Bicalho et al. [39], PNAE management performance indicators can facilitate monitoring and evaluation initiatives, and, in addition to guiding government action, can facilitate social control to be performed by CAE. In addition, other authors reflect on the interconnection of the performance of various programs and the effect of such interconnection with social inequities and food [40,41].

Also, school meal programs are of particular interest for improving public diet because they reach children at a population scale across socioeconomic classes and for over a decade of their lives, and because the food habits of children are more malleable than those of adults [42]. This reinforces the importance of school meals in encouraging healthy eating and health promotion.

The PNAE proposal also contributes to food sustainability by encouraging the inclusion of family farming products in schoolchildren’s diets, thereby increasing the consumption of fruits and vegetables, and promoting food and nutritional education. Indeed, school meals can be considered as a tool for promoting healthy and sustainable food behaviors within the population in the long term [43].

Promoting healthy eating habits within school meals is an essential tool for combating obesity in children and adolescents, in line with the Global Strategy on Diet, Physical Activity and Health [44] which warns of the development of diseases resulting from unhealthy eating habits, in addition to studies that indicate the importance of nutritional education in the school environment, as a path to the healthy development of children and adolescents [45].

As demonstrated in the code cloud, achieving the PNAE objectives depends on the successful performance of social control, and strengthening the CAE can be achieved based on education aimed at understanding the role of the CAE through partnerships with public entities such as Universities, Federal Institutes, Public Prosecutors, etc., to demand effective actions from the actors involved in the Program, to deliver the results provided for in the Legislation [46].

Examining the results of this work alongside those of other countries and entities within the Federation (Brazil) prompts reflection on the importance of developing countries, such as Brazil, in establishing and maintaining social control within their government structures.

## 5. Conclusions

The analyses indicate that, for the effective exercise of the right, social control is essential. Despite this, the FNDE lacks monitoring mechanisms that can quickly detect evidence of non-compliance with the Program, hence the importance of social control in monitoring the PNAE, with the CAE being the body responsible for verifying compliance with the Program’s objectives. When active, the CAE can promote social control, allowing the adoption of timely measures to correct the Program’s course and contribute to achieving its objectives.

This is the paradigm of all Public Policies, as they depend on a series of other guarantees from the State for their financing and implementation, such as qualified managers, adequate financial support commensurate with the program’s objectives, or even necessary infrastructure. These government mechanisms are part of the scope of the Policies and are, in fact, essential for the development of any Policy. Such management mechanisms must also provide means of evaluating these policies to ensure constant improvement and correction of any distortions.

Evaluating Public Policies is crucial for verifying the actual fulfillment of guaranteed rights, correcting directions, and ensuring that the dietary attention provided in the PNAE meets all political and technical guidelines, thereby guaranteeing students access to food that promotes healthy habits and health.

The results of this study aim to contribute to researchers working on public policies and food and nutrition security, based on the analysis of key documents addressing food and nutrition security, considering the main FNS program in Brazilian schools, the PNAE. Through this critical qualitative analysis, we found that the applicability of the broad set of regulations aimed at guaranteeing human rights will be more effective if there is public participation through social oversight. This analysis directly contributes to the fact that both researchers and actors involved in the development of food security policies in Brazil and around the world can emphasize the importance of methods for applying rights, to involve the entire society in the implementation of the proposed policies, aiming to achieve the elimination of inequalities and the Human Right to Adequate Food and health promotion through healthy eating.

## Figures and Tables

**Table 2:** Codes and definitions adopted a priori.

Codes	Adopted Definition
Proper nutrition	It is the realization of a basic human right, with the guarantee of permanent and regular access, in a socially fair manner, to a dietary practice that is appropriate to the biological and social aspects of individuals, according to their life cycle and special dietary needs, based on local traditional references. It must comply with the principles of: • Variety, • Balance, • Moderation, • Pleasure (flavor), • gender and ethnic dimensions, and • Environmentally sustainable forms of production, free from physical, chemical, biological contaminants and genetically modified organisms. of the Program. The dimensions of adequate food are sanitary quality, nutritional adequacy, diversity, respect for and appreciation of national and regional food culture, access to financial resources or natural resources such as land and water, absence of pesticides and genetically modified organisms and access to information [28]
Organic food	Products are produced in an organic production environment, where agroecological principles are used as the basis of the production process, which includes the responsible use of soil, water, air and other natural resources, respecting social and cultural relations [22]
Social/democratic control	It involves monitoring and surveilling public policies, allocating the public budget, and ensuring government actions by society to protect rights and effectively implement public policies. It is the opportunity for actors and social segments to intervene in public policy decisions to contribute to the State’s effective action in guaranteeing all human rights [29]
Audit (positive and negative)	Systematic process of obtaining and objectively evaluating audit evidence to determine whether the information or actual conditions of an object are in accordance with the applicable criteria; systematic, documented and independent process of objectively evaluating a situation (or condition) to determine the extent to which the applicable criteria are met, obtaining evidence regarding compliance and reporting the results of the evaluation to predetermined recipients—Inspection instrument used by the Federal Court of Auditors-TCU to examine the legality and legitimacy of the management acts of those responsible under its jurisdiction, with regard to the accounting, financial, budgetary and patrimonial aspects; to evaluate the performance of the bodies and entities under its jurisdiction, as well as of the government systems, programs, projects and activities, with regard to the aspects of economy, efficiency and effectiveness of the acts performed; to support the assessment of the acts subject to registration [23,29].
Menu	Operational tool that lists foods intended to meet individual and collective nutritional needs, discriminating foods by preparation, quantity per capita, for energy, carbohydrates, proteins, lipids, vitamins and minerals and according to the labeling standard (it is the official communication made by the manager, through a newspaper, website or in the form of a widely circulated notice board for public knowledge of the demands for the acquisition of foodstuffs from family farming for school meals [30].
Public call for purchase	Official communication was made by the manager through a newspaper, website, or on a widely circulated notice board, for public knowledge of the demands for food products from family farming for school meals [30]
Food control and regulation	Food quality monitoring must consider health aspects, such as microbiological and toxicological aspects, as well as nutritional profile aspects, including macro- and micronutrient levels, in conjunction with mandatory food fortification strategies and the reformulation of processed food nutritional profiles to reduce fats, sugars, and sodium [30].
Complaint	Constitutional prerogative of any citizen, political party, association, or union to report irregularities or illegalities (illegal acts) to the courts in matters within their jurisdiction, carried out by an administrator or person responsible under their authority [31]
HRAF	The right to adequate food is a human right inherent to all people to have regular, permanent and unrestricted access, either directly or through financial acquisitions, to safe and healthy food, in adequate and sufficient quantity and quality, corresponding to the cultural traditions of their people and which guarantees a life free from fear, dignified and full in the physical and mental, individual and collective dimensions [32].
Economy	Minimize the costs of resources used in carrying out an activity without compromising quality standards. Refers to the ability of an institution to effectively manage the resources made available to it [33].
Food and Nutrition Education—EAN	For the PNAE, EAN is considered to be the set of training actions, of continuous and permanent practice, transdisciplinary, intersectoral and multi-professional, which aims to stimulate the voluntary adoption of healthy dietary practices and choices that contribute to learning, the health status of the student and the quality of life of the individual [16].
Effectiveness	Degree of achievement of programmed goals in terms of products (goods or services) in a given period, regardless of the costs involved. The concept of effectiveness concerns the management’s ability to meet immediate objectives, translated into production or service goals, that is, the ability to provide goods or services in accordance with what was planned [33].
Efficiency	Relationship between the products (goods or services) generated by an activity and the costs of the inputs used to produce them in a given period, while maintaining quality standards. It refers to the effort required to transform inputs into products. It can be examined from two perspectives: minimizing the total cost or the means necessary to obtain the same quantity and quality of product, or optimizing the combination of inputs to maximize the product when the total cost is previously fixed [33].
Oversight	Power-duty of surveillance, examination, or verification assigned by law to a public body, entity, or agent. The accounting, financial, budgetary, operational, and asset supervision of the Union is exercised by the National Congress, through external control, with the assistance of the Federal Court of Auditors. The Federal Court of Auditors uses the inspection instruments provided for in its Internal Regulations to fulfill this duty [33].
Technical Management	They include the technical duties of nutrition professionals within the scope of the PNAE, such as planning the PNAE and preparing menus, Technical Preparation Files (TPFs), nutritional diagnosis, Food and Nutritional Education Activities, and other duties defined in CFN Resolution No. 465 of 2010 [22].
Irregularity	Failure to render accounts; practice of an illegal, illegitimate or uneconomical management act, or violation of the legal or regulatory norm of an accounting, financial, budgetary, operational or patrimonial nature; damage to the public treasury resulting from an illegitimate or uneconomical management act; embezzlement or diversion of public funds, assets or values; failure to comply with a determination of which the responsible party was aware, made in the process of taking or rendering accounts; violations of the principles of public administration [34].
Qualified Nutritionist	Professionals hold a Professional Identity Card issued by the Regional Council of Nutritionists (CRN) and are regularly registered with the CRN, in accordance with current legislation [22].
Planning	Planning involves deciding in advance what to do, how to do it, when to do it, and who should do it. Planning covers the space between where we are and where we want to go. It makes the occurrence of events that would otherwise not happen possible. Although the exact future cannot be predicted, and uncontrollable factors can interfere with the best-laid plans, without planning, events are left entirely to chance. Planning is a demanding intellectual process; it requires a conscious determination of alternatives for action and the grounding of decisions in purposes, knowledge, and careful estimates [35].
Public policy	A strategy of thought-out, planned, and evaluated action, guided by collective rationality, in which both the State and society play active roles, continuously and simultaneously implying state intervention, involving different actors (governmental and non-governmental), whether through demands, support, or assistance, or democratic control [36].
Accountability	The accountability to be carried out by the Executing Entity, according to Resolution CD/FNDE No. 2 of 2012 and its amendments, consists of proof of achievement of the object and objective of the Program, the correct application of the financial resources transferred in each fiscal year and compliance with the rules relating to the technical and financial aspects of the execution of the Program [37].
Promotion of adequate and healthy eating (PAAS)	Adequate and healthy nutrition is understood as the dietary practice appropriate to the biological and sociocultural aspects of individuals, as well as to the sustainable use of the environment. In other words, it must follow the needs of each phase of the life course and with special dietary needs; referenced by food culture and the dimensions of gender, race and ethnicity; accessible from a physical and financial point of view; harmonious in quantity and quality; based on appropriate and sustainable production practices with minimum quantities of physical, chemical and biological contaminants [36].
Technical staff	The Technical Staff will comprise qualified nutritionists who will carry out the activities defined in this Resolution and other standards issued by the CFN, following the FNDE standards, under the coordination and supervision of the technical manager, assuming joint responsibility with them [22].
Food quality	The food dimension refers to the production and availability of food, which must be: • Sufficient and adequate to meet the population’s demand, in terms of quantity and quality; • Stable and continuous to guarantee permanent supply, neutralizing seasonal fluctuations; • Autonomous to achieve national self-sufficiency in basic foods; • Equitable to guarantee universal access to adequate nutritional needs, to maintain or restore health at different stages of the life course and in different population groups; • Sustainable from an agroecological, social, economic and cultural point of view, to ensure the FNS of future generations [9].
Nutritional quality	The nutritional dimension incorporates the relationship between human beings and food, involving: • Availability of healthy foods; • Preparation of food using techniques that preserve its nutritional and health value; • Adequate and healthy food consumption for each phase of the life cycle; • Conditions for promoting health, hygiene and a healthy lifestyle to improve and ensure the adequate biological use of the food consumed; • Conditions for promoting care for one’s own health, the health of the family and the community; • The right to health, with access to health services guaranteed in a timely and effective manner; • Prevention and control of determinants that interfere with health and nutrition, such as psychosocial, economic, cultural and environmental conditions; • Good opportunities for personal and social development in the place where one lives and works [9].
Representation	Prerogative of bodies, entities or persons entitled to present to the Court irregularities or illegalities committed by an administrator or person responsible under their jurisdiction, in matters within their competence, written in clear and objective language, containing the legible name, qualification and address of the representative, and accompanied by evidence concerning the irregularity or illegality represented [33].
Technical responsibility	Legal attribution is given to qualified nutritionists, as analyzed by the CRN, who are professionals responsible for planning, coordinating, directing, supervising, and evaluating activities related to food and nutrition within legal entities [22].
Food and Nutrition Security	It is understood as the realization of everyone’s right to regular and permanent access to quality food, in sufficient quantity, without compromising access to other essential needs, based on health-promoting eating practices that respect cultural diversity and are environmentally, culturally, economically, and socially sustainable [17].
Sustainability	Principle closely linked to the social responsibility of organizations, whose purpose is to obtain and maintain suitable conditions for society as a whole and the planet through sustainable development when dealing with social, economic and environmental aspects, so that the use of resources to satisfy present needs cannot compromise the satisfaction of the needs of future generations [29]
Acceptability test	A set of scientifically recognized methodological procedures designed to measure the acceptability index of food offered to schoolchildren [28].

Source: Prepared by the authors based on the indicated references.

**Table 3:** Recording Units included during the coding process.

Recording Unit	Adopted Definition
PNAE Financing	Administrative measures that favor or not the financial contribution destined for the School Feeding Program, both by the FNDE and by the DF government.
Inefficiency	Aspects related to the lack of efficiency of actions that result in harm to public management.
Technical justification for food acquisition	The technical basis that presents all the variables resulting from a given future action by the public manager and that justifies the need for adjustments, especially regarding the specification of products to be acquired by the public administration. Document prepared by professionals who are technically qualified for this purpose.
Planning Positive and Negative	Planning is deciding in advance what to do, how to do it, when to do it, and who should do it. Planning covers the space between where we are and where we want to go. It makes possible the occurrence of events that would otherwise not happen. Although the exact future cannot be predicted, and uncontrollable factors may interfere with the best-laid plans, without planning, events are left entirely to chance. Planning is a demanding intellectual process; it requires a conscious determination of alternatives for action and the grounding of decisions in purposes, knowledge, and careful estimates [36].
Reform	Indication of the need for reform in the infrastructure of school kitchens.
Monitoring	Monitor actions developed within the scope of the PNAE, as well as investments, expenses, food purchases, and infrastructure, and prepare substantiated reports that support public manager decisions.
Non-performance of contract	Failure to comply with the provisions of the contract.
Overprice	The price charged exceeds the normal or listed price.

Source: Prepared by the authors.

## Data Availability

The original contributions presented in this study are included in the article. Further inquiries can be directed to the corresponding author.
